# Long‐Term Albumin Therapy in Cirrhosis With Ascites: Not an Answer yet

**DOI:** 10.1002/ueg2.70278

**Published:** 2026-08-18

**Authors:** Adrià Juanola, Pere Ginès

**Affiliations:** ^1^ Hospital Clinic de Barcelona Barcelona Spain; ^2^ Fundació de Recerca Clinic Barcelona—Institut d'Investigacions Biomediques August Pi i Sunyer Barcelona Spain; ^3^ Centro de Investigación Biomédica en Red Enfermedades Hepáticas y Digestivas Madrid Spain; ^4^ Universitat de Barcelona Facultat de Medicina i Ciències de la Salut Barcelona Spain

Albumin is the most abundant plasma protein and plays a central role in the regulation of intravascular volume through its contribution to plasma oncotic pressure. Beyond its hemodynamic effects, albumin exerts important immunomodulatory and antioxidant functions and participates in lipid transport and metabolism [1]. Because albumin is synthesized exclusively by the liver, patients with cirrhosis experience a progressive decline in serum albumin concentration as liver function deteriorates. Consequently, serum albumin is one of the strongest prognostic markers in cirrhosis and constitutes a key component of contemporary prognostic models [2, 3, 4].

Traditionally, reduced plasma oncotic pressure has been considered a major determinant of the accumulation of fluid in the extravascular compartment, leading to the development of ascites and peripheral edema, hallmarks of advanced cirrhosis. However, it is now recognized that the pathophysiology of fluid retention in cirrhosis is far more complex than hypoalbuminemia alone and involves profound circulatory and neurohumoral disturbances [5].

Against this background, several early studies explored whether repeated infusions of human albumin could modify the natural history of decompensated cirrhosis [6]. Although these initial investigations yielded largely disappointing results, interest in albumin therapy was revived by the ANSWER trial, a multicenter, open‐label, randomized study conducted in Italy [7]. This trial enroled patients with decompensated cirrhosis and uncomplicated ascites requiring chronic diuretic therapy and compared standard medical treatment alone with standard treatment plus long‐term albumin administration (40 g twice weekly for the first 2 weeks, followed by 40 g weekly for up to 18 months). Albumin therapy was associated with a reduced incidence of several major complications of cirrhosis, including refractory ascites, spontaneous bacterial peritonitis and other infections, grade III‐IV hepatic encephalopathy, and acute kidney injury/hepatorenal syndrome. Importantly, albumin therapy was also associated with a significant survival benefit, reducing overall 18‐month mortality by 38% compared with standard medical therapy alone (HR 0.62, 95% CI 0.40–0.95). Nevertheless, several limitations of this study merit consideration. The open‐label design could have favoured patients assigned to albumin because of the closer clinical surveillance associated with weekly hospital visits. In addition, follow‐up was interrupted in patients who required three or more large‐volume paracenteses per month or underwent TIPS placement, circumstances that occurred in a substantial number of participants. Although these findings have not yet led to universal endorsement of long‐term albumin therapy in international guidelines, they have generated considerable interest and debate within the hepatology community.

In this issue of *United European Gastroenterology Journal*, Pompilii et al. [8] provide new insights through a post hoc analysis of the ANSWER trial examining whether baseline serum albumin levels influence the efficacy of long‐term albumin therapy. Their main finding is that the benefits of albumin administration appear to be greatest in patients with the lowest serum albumin concentrations at treatment initiation. Using cubic spline analyses, the authors observed a survival benefit at 18 months among patients with baseline albumin levels below 32 g/L who received albumin, whereas no significant survival advantage was identified in those with albumin concentrations above this threshold. Interestingly, albumin therapy continued to reduce the occurrence of several cirrhosis‐related complications even in patients with higher baseline albumin levels. Analysis of crude mortality showed that the most clinically relevant difference in mortality occurred at the threshold of 28.4 g/L (see table 2 of the manuscript).

These findings should be interpreted cautiously given the inherent limitations of post hoc analyses. Nonetheless, they offer a biologically plausible explanation for the heterogeneous response to albumin therapy and suggest that patients with more profound hypoalbuminemia may derive the greatest benefit from long‐term treatment. Conversely, among patients whose albumin levels are closer to the normal range, the therapeutic effect appears to be less pronounced and may be largely restricted to the prevention of complications rather than improvement in survival.

Should these results be incorporated into clinical practice? At present, our answer is probably no. Although the study by Pompilii et al. [8] advances our understanding of which patients may more likely benefit from albumin therapy, important uncertainties remain. Notably, the survival benefit observed in the ANSWER trial has not been consistently reproduced in other studies. In contrast to ANSWER, the double‐blind, placebo‐controlled MACHT trial [9] failed to demonstrate improvements in either survival or the incidence of cirrhosis‐related complications among patients receiving albumin associated with midodrine versus those receiving placebo, yet the albumin dose given in that study was lower. More recently, the PRECIOSA study [10], reported in abstract form, did not meet its primary endpoint of transplant‐free survival despite using a broadly similar therapeutic strategy to that of the ANSWER trial.

Taken together, the available evidence suggests that long‐term albumin administration remains a promising but not yet fully established therapeutic intervention in decompensated cirrhosis. The findings reported by Pompilii et al. [8] are encouraging and help refine the profile of patients most likely to benefit from treatment. However, before baseline serum albumin concentration can be used to guide treatment decisions, the survival benefit reported in ANSWER should ideally be confirmed in a new large, multicenter randomized controlled trial specifically designed to address the remaining uncertainties surrounding long‐term albumin therapy.

## Conflicts of Interest

P.G. has received research funding from Grifols. P.G. has consulted or attended advisory boards for SeaBeLife, Merck, Sharp and Dohme (MSD), Ocelot Bio, Behring, Roche Diagnostics International, Alpha Sigma, Boehringer Ingelheim, Astra‐Zeneca and Sysmex and received speaking fees from Pfizer and Grifols. A.J. reports speaking fees from Grifols.

## Data Availability

The authors have nothing to report.
